# Consensus in determining the resectability of locally progressed pancreatic ductal adenocarcinoma – results of the Conko-007 multicenter trial

**DOI:** 10.1186/s12885-019-6148-5

**Published:** 2019-10-22

**Authors:** U. A. Wittel, D. Lubgan, M. Ghadimi, O. Belyaev, W. Uhl, W. O. Bechstein, R. Grützmann, W. M. Hohenberger, A. Schmid, L. Jacobasch, R. S. Croner, A. Reinacher-Schick, U. T. Hopt, A. Pirkl, H. Oettle, R. Fietkau, H. Golcher

**Affiliations:** 1 Department for General- und Visceral Surgery, Medical Center and Faculty of Medicine University of Freiburg, Hugstetter Straße 55, 79106 Freiburg, Germany; 2 Department of Radiation Oncology, Friedrich-Alexander-Universität Erlangen-Nürnberg (FAU), Erlangen, Germany; 3 Department of General, Visceral and Pediatric Surgery, Medical Center Georg-August-University Göttingen, Göttingen, Germany; 4 Department of Surgery, St. Josef Hospital Ruhr-University Bochum, Bochum, Germany; 5 Department of General and Visceral Surgery, Frankfurt University Hospital and Clinics, Frankfurt, Germany; 6 Department of Surgery, Friedrich-Alexander-Universität Erlangen-Nürnberg (FAU), Erlangen, Germany; 7 Department of Radiology, Friedrich-Alexander-Universität Erlangen-Nürnberg (FAU), Erlangen, Germany; 8 Private Practice, Hematology/Oncology, Dresden, Germany; 9 Department of Surgery, University Hospital Magdeburg, Magdeburg, Germany; 10 Department for Hematology, Oncology and Palliative Care, St Josef-Hospital, Ruhr-University Bochum, Bochum, Germany; 11 Medical Centre for Information and Communication Technology, Friedrich-Alexander-Universität Erlangen-Nürnberg (FAU), Erlangen, Germany; 12 Outpatient Department Hematology/Oncology, Friedrichshafen, Germany

**Keywords:** Pancreatic cancer, Determination of resectability, Locally advanced, Borderline resectable, Prospective randomized multicenter trial

## Abstract

**Background:**

One critical step in the therapy of patients with localized pancreatic cancer is the determination of local resectability. The decision between primary surgery versus upfront local or systemic cancer therapy seems especially to differ between pancreatic cancer centers. In our cohort study, we analyzed the independent judgement of resectability of five experienced high volume pancreatic surgeons in 200 consecutive patients with borderline resectable or locally advanced pancreatic cancer.

**Methods:**

Pretherapeutic CT or MRI scans of 200 consecutive patients with borderline resectable or locally advanced pancreatic cancer were evaluated by 5 independent pancreatic surgeons. Resectability and the degree of abutment of the tumor to the venous and arterial structures adjacent to the pancreas were reported. Interrater reliability and dispersion indices were compared.

**Results:**

One hundred ninety-four CT scans and 6 MRI scans were evaluated and all parameters were evaluated by all surgeons in 133 (66.5%) cases. Low agreement was observed for tumor infiltration of venous structures (κ = 0.265 and κ = 0.285) while good agreement was achieved for the abutment of the tumor to arterial structures (interrater reliability celiac trunk κ = 0.708 *P* < 0.001). In patients with vascular tumor contact indicating locally advanced disease, surgeons highly agreed on unresectability, but in patients with vascular tumor abutment consistent with borderline resectable disease, the judgement of resectability was less uniform (dispersion index locally advanced vs. borderline resectable *p* < 0.05).

**Conclusion:**

Excellent agreement between surgeons exists in determining the presence of arterial abutment and locally advanced pancreatic cancer. The determination of resectability in borderline resectable patients is influenced by additional subjective factors.

**Trial registration:**

EudraCT:2009-014476-21 (2013-02-22) and NCT01827553 (2013-04-09).

## Background

Only 15–20% of patients diagnosed with pancreatic cancer present with resectable disease [1, 2]. These patients have a chance for cure and benefit from surgical resection [3, 4]. In 50–55%, pancreatic cancer has already metastasized, but in the remaining patients the tumor cannot be removed surgically due to local disease progression [5, 6]. This is due to invasion or contact of the tumor to peripancreatic vessels [7]. Several guidelines have been established to discriminate resectable and borderline resectable from locally advanced cases [8–12]. According to ISGPS definitions, the tumor contact to the celiac trunk, more than 180° abutment to the superior mesenteric artery, infiltration of the inferior vena cava, unreconstructable superior mesenteric vein or occlusion of the portal vein, or aortic invasion or encasement are considered signs of locally advanced pancreatic cancer [12]. Borderline resectable patients are defined by the involvement of the superior mesenteric vein or portal vein allowing safe resection and reconstruction. These patients may also display gastroduodenal artery encasement including possible short segment encasement or direct abutment of the hepatic artery and less than 180° abutment of the superior mesenteric artery [8].

Despite these definitions, many surgeons disagree on a number of details even in patients not treated with neoadjuvant therapy. The involvement of the venous confluence undercrossing the pancreatic neck is considered resectable by many surgeons, as long as the reconstruction of the venous axis can be performed. Similarly, abutment of the hepatic artery of more than 180° is considered locally advanced by many surgeons, since arterial reconstructions are associated with high perioperative mortality and poor oncologic outcome [13].

That fact that different definitions of resectability can be applied indicates that the judgement of resectability may also be influenced by subjective factors. Despite the fact that previous reports have already indicated variability in the judgement of tumor contact to defined anatomic structures, the consensus in judging resectability by pancreatic surgeons has not been evaluated in a large cohort of locally advanced or borderline resectable patients [14].

**Fig. 1:**
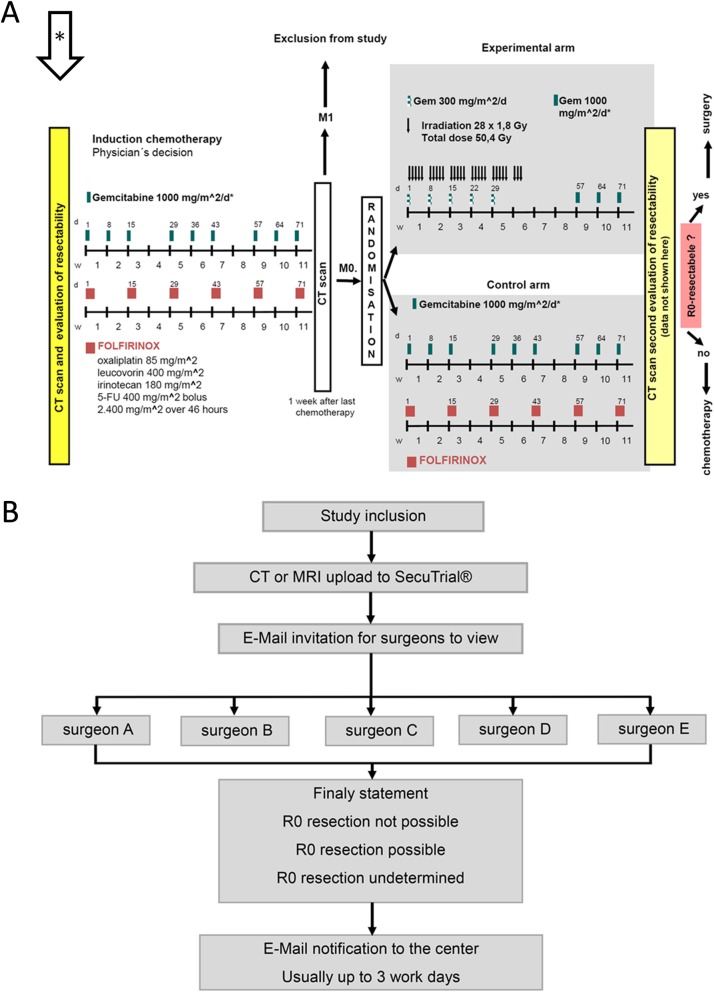
Treatment and Procedure of Evaluation of Pretherapeutic Radiographs. **a** Schematic view of the treatment algorithm of the Conko-007 trial. Patients will be restaged after induction chemotherapy and if no distant metastasis is present randomized to the two treatment arms. After 6 months of treatment, final evaluation is performed and surgical resection is attempted. Radiographs of the initial staging prior to neoadjuvant chemotherapy were analyzed (arrow with asterisk) **b** Flowchart for the evaluation of the pretherapeutic radiographs. After upload of the abdominal MRI or CT scans by the trial center, the evaluating surgeons were contacted by e-mail and requested to evaluate the radiographs within the next 3 workdays

The prospective, randomized, multicenter phase III trial CONKO-007 (EudraCT: 2009–014476-21, NCT01827553) examines the value of radiation therapy in patients with locally advanced or borderline resectable pancreatic cancer. The study protocol combines systemic treatment and chemoradiotherapy (Fig. 1a) which may be associated with a survival benefit in patients with locally advanced PDAC, especially if secondary resectability can be achieved [15–17]. To confirm the judgement of local resectability by the enrolling center, CT or MRI scans of the patients enrolled are additionally reevaluated by a panel of 5 experienced pancreatic surgeons in an independent and prospective manner. The evaluation is either undertaken by one experienced pancreatic surgeon of a high volume center or in one center by an interdisciplinary team consisting of an experienced pancreatic surgeon, radiologist and oncologist. Vascular involvement is evaluated and reported. Finally, a non-binding overall judgement of resectability is reported to the enrolling center (Fig. 1b).

With our analysis, we examined and compared the judgement obtained in the first 200 consecutive patients enrolled in the CONKO-007 trial and identified critical factors in evaluating tumor abutment and resectability in patients with borderline and locally advanced pancreatic cancer.

## Methods

### Study design and patient acquisition

The CONKO-007 trial examines the effectiveness of chemoradiation compared to chemotherapy alone after induction chemotherapy with 3 cycles of gemcitabine or 6 cycles of FOLFIRINOX (Fig. 1a).

Eligible patients were aged 18 years or older with histologically-confirmed non-resectable adenocarcinoma of the pancreas without distant metastasis according to CT-imaging of thorax and abdomen. ECOG-performance status was equal or less than two. Each patient provided written informed consent before participating in the study.

The trial was conducted following ICH-GCP guidelines, approved by the central Ethics Committee (University Hospital Erlangen, 322_12AZ) and by the Federal Institute for Drugs and Medical Devices (BfArM, 4,038,763). The trial registration (EudraCT: 2009–014476-21, NCT01827553) was obtained prior to recruitment. Patients were recruited at 52 German cancer centers.

The data concerning pretherapeutic radiographs collected prospectively from the first 200 patients included in the trial were evaluated in this analysis. 50% of the patients were consecutively enrolled at 6 cancer centers: Erlangen, Magdeburg, Göttingen, Dresden, Freiburg, Bochum (> 10 patients per center) and 50% at the remaining centers (1–9 patients per center) between 04/2013 and 07/2016. The sequence of evaluation and documentation is displayed in Fig. 1a.

The CONKO-007 study network uses a GCP-certified commercially available clinical trials management system SecuTrial® [18]. After patient’s enrollment, the participating centers uploaded CT or MR scans performed prior to therapy in the globally standardized DICOM format. One hundred ninety scans were sent as scheduled in the study protocol. The missing 10 scans were handed over at the monitoring visits. SecuTrial® pseudonymized the DICOM images during upload. Once the upload was completed, 5 leading pancreatic surgeons automatically got an email containing the invitation to view the images.

The initial radiographs taken prior to enrollment were either reviewed in a web based fashion online or downloaded and analyzed in a DICOM viewer. In 4 high volume centers for pancreatic surgery, experienced pancreatic surgeons evaluated the uploaded pictures blinded except for age, sex and study site. In one case, the CT or MR images were presented to the interdisciplinary tumor board and simultaneously evaluated by a radiologist and a surgeon. The observers were aware of the judgement of the enrolling center.

The following items were documented: Suspected liver metastasis, suspected peritoneal metastasis, distance between the tumor and vascular structure of more than 1 mm, tumor contact of less than 180°, tumor contact of more than 180° to the vascular structures. Furthermore, a category “cannot be defined” was available. The vascular structures evaluated were celiac trunk, common hepatic artery, superior mesenteric artery, branches of the jejunal artery, superior mesenteric vein, and portal vein. Finally, resectability was evaluated either as locally advanced, complete R0 resection possible, and R0 resection undetermined. The statement of the panel concerning resectability (R0 possible, not possible or undetermined) did not influence the sequence of treatment but was provided automatically to the participating centers by email, usually within 3 working days.

### Statistical analysis

Analysis of parameters associated with local resectability was performed using SPSS (IBM Version 23.0, IBM Armonk, NY, USA) in conjunction with Excel (Microsoft, Redmond, WA). Significance was determined by Chi-square test with post-hoc analysis by cellwise adjusted residual analysis in two- way contingency tables according to Garcia-Perez [19]. Multiple comparisons were accounted for by Bonferroni correction. Interobserver agreement was calculated by the estimation of Fleiss-kappa. A κ of below 0.199 indicates poor agreement, 0.200–0.399 indicates fair agreement, 0.400–0.599 indicates moderate agreement, 0.600–0.799 indicates strong agreement, and more than 0.800 indicates a very strong and almost perfect agreement. The dispersion index was calculated according to Loether and MacTavish [20] and compared by Kruskal-Wallis-Test for independent samples followed by a Bonferroni post hoc test. A dispersion index of 0 indicates a perfect match of all 5 examiners.

## Results

One hundred ninety-four CT and six MRI scans were evaluated by 5 independent surgeons. Two hundred cases were evaluated and a judgement was available in 943 instances (94.3%). In 133 cases, all parameters were judged by all surgeons. In 60 further cases, only one surgeon was unable to perform the judgement for one parameter. The quality of the radiographs was good enough that in only 7 cases two or more surgeons were unable to perform their judgement on one of the parameters questioned. This did not have an impact on the heterogeneity of judgement. No differences in the results were detected between CT and MRI cases.

### Resectability is judged differently by experienced surgeons

When five independent pancreatic surgeons viewed the cases and judged the possibility for complete tumor resection, significant differences in the judgements were observed (Table 1). While surgeons A, B, and C found it impossible in 72.3–74.7% of the cases to achieve complete tumor resection according to the provided radiographs, surgeons D and E found it impossible in 88.5 and 91.4%. This was not only due to a lower number of patients with borderline resectability but also due to significantly (*p* < 0.05) more patients considered resectable by the other surgeons (Table 1).

**Table 1:** Judgement of resectability by 5 independent surgeons

	Surgeon A		Surgeon B		Surgeon C		Surgeon D		Surgeon E	
	[n]	[%]	[n]	[%]	[n]	[%]	[n]	[%]	[n]	[%]
R0 Resection Possible	6	3.1	16	8.1	12	7.5	3	1.6	3*	1.5
R0 Resection Questionable	43	22.2	35	17.7	32	20.1	19	9.9	14	7.1
R0 Resection Impossible	145	74.7	147	74.2	115	72.3	170	88.5	181	91.4
Cases without evaluation of resectability	6		2		41		8		2	

Surgeons evaluated the local resectability of progressed pancreatic cancer. They classified the case (n) into R0 resection possible for resectable cases, R0 resection impossible for locally advanced cases and R0 resection questionable for borderline resectable cases. Significant deviations in judgement were observed with surgeon E matching significantly less cases as resectable (χ^2^ frequency distribution with post hoc analysis by cellwise adjusted residuals * *P* < 0.05)

### Low agreement in venous tumor abutment

Since resectability is defined by the technical possibility to dissect the tumor from the peripancreatic arteries or to be able to resect and reconstruct the venous confluens, we analyzed the parameters on which the judgement of resectability is based. The consensus between the individual surgeons was dependent on the vessels assessed (Table 2). The highest conformity in the assessments of the individual surgeons was achieved for the contact of the tumor to the celiac trunk. The judgement of tumor contact to venous structures and the jejunal branches of the mesenteric artery appeared to be more complex, since the conformity in assessment was lower with a κ = 0.285 for tumor contact to the portal vein and κ = 0.265 to the superior mesenteric vein.

**Table 2:** Agreement in the assessment of tumor contact to vascular structures

Basis *n* = 200 (100%)		Celiac trunk [n; %; κ]	Common hepatic artery [n; %; κ]	Superior mesenteric artery [n; %; κ]	Jejunal artery [n; %; κ]	Portal vein [n; %; κ]	Superior mesenteric vein [n; %; κ]
Identical assessment <> 180° involvement	N	88	72	82	63	59	57
	%	44.0%	36.0%	41.0%	31.5%	29.5%	27.5%
	κ	0.526	0.445	0.477	0.209	0.285	0.265
Identical assessment with any vascular involvement	N	133	124	134	129	84	84
	%	66.5%	62.0%	67.0%	64.5%	42.0%	42.0%
	κ	0.708	0.639	0.623	0.512	0.256	0.336

The consensus in the assessment of tumor contact to the large peripancreatic vessels was determined by calculating the interrater reliability for each item. The interrater reliability represents the agreement between the 5 observers with 1 indicating a perfect match. When the grading of tumor contact included the degree of tumor contact, agreement was reached in only 57–88 of the cases (27.5–44.0%). When tumor contact was graded independent of the degree of tumor contact, the agreement increased to 42.0–67.0% of the cases showing strong agreement

### Variability increases by estimating the degree of arterial tumor abutment

When the concordance between the judgement of tumor contact of less and more than 180° was examined, it also became evident that subdividing the degree of tumor contact into two classes introduced additional variation and reduced conformity (Table 2). This indicated that differentiation between the degrees of tumor contact increased the subjectivity of judgement. By omitting the degree of tumor contact and merging the two categories, the interrater agreement was increased for the celiac trunk, the common hepatic artery and the mesenteric artery, while moderate agreement was obtained for the abutment of jejunal branches. The discrepancies in the judgement of portal vein and superior mesenteric vein affection remained unaltered, indicating that the evaluation of the tumor contact to the large peripancreatic veins is not feasible and is a substantial source of subjectivity.

### Surgeons adhere to ISGPS recommendations

To further investigate the influence of tumor abutment in analogy to ISPGS guidelines on the expectance of complete tumor resection, the tumor abutment observed by the surgeons was translated into resectability according to ISGPS recommendations and compared to the evaluation of resectability provided by the same surgeon (Table 3). In the vast majority of cases, the calculated resectability matched the judgment of the observer (72.9–83.9%). Despite tumor abutment indicating locally advanced disease, 8.3–21.4% of the cases were still considered R0 resectable with significant differences between the individual surgeons. Locally advanced or borderline resectable tumors involved arterial affection in most cases (92.7–98.4%), indicating that infiltration and occlusion of the portovenous axis without arterial abutment occurs in less than 10% of locally advanced PDAC.

**Table 3:** Calculated resectability vs. evaluated resectability

		Surgeon A	Surgeon B	Surgeon C	Surgeon D	Surgeon E
Agreement between Surgeon and ISGP		76.1%	83.4%	74.1%	72.9%	79.8%
Disagreement		23.9%	16.6%	25.9%	27.1%	20.2%
*Surgeon*	*ISGP*					
Resectable	Locally advanced	1.0%	1.0%	1.3%	2.6%	4.6%
Resectable	Borderline resectable	7.3%	3.5%	13.9%	18.8%	9.6%
Locally advanced	Resectable	0.5%	2.5%	0%	0%	0%
Locally advanced	Borderline resectable	0%	0%	1.9%	0.5%	0%
Undetermined	Resectable	6.8%	2.5%	2.5%	1.6%	2.0%
Undetermined	Locally advanced	8.3%	7.1%	6.3%	3.6%	4.0%

Resectability was calculated from the single items assessed by the 5 surgeons according to ISGPS recommendations. The resectability calculated from the assessment of tumor abutment to peripancreatic vascular structures was compared to the judgement of resectability given by the evaluating surgeon

### Assessment of resectability is less homogeneous in borderline resectable patients than in locally advanced patients

**Fig. 2:**
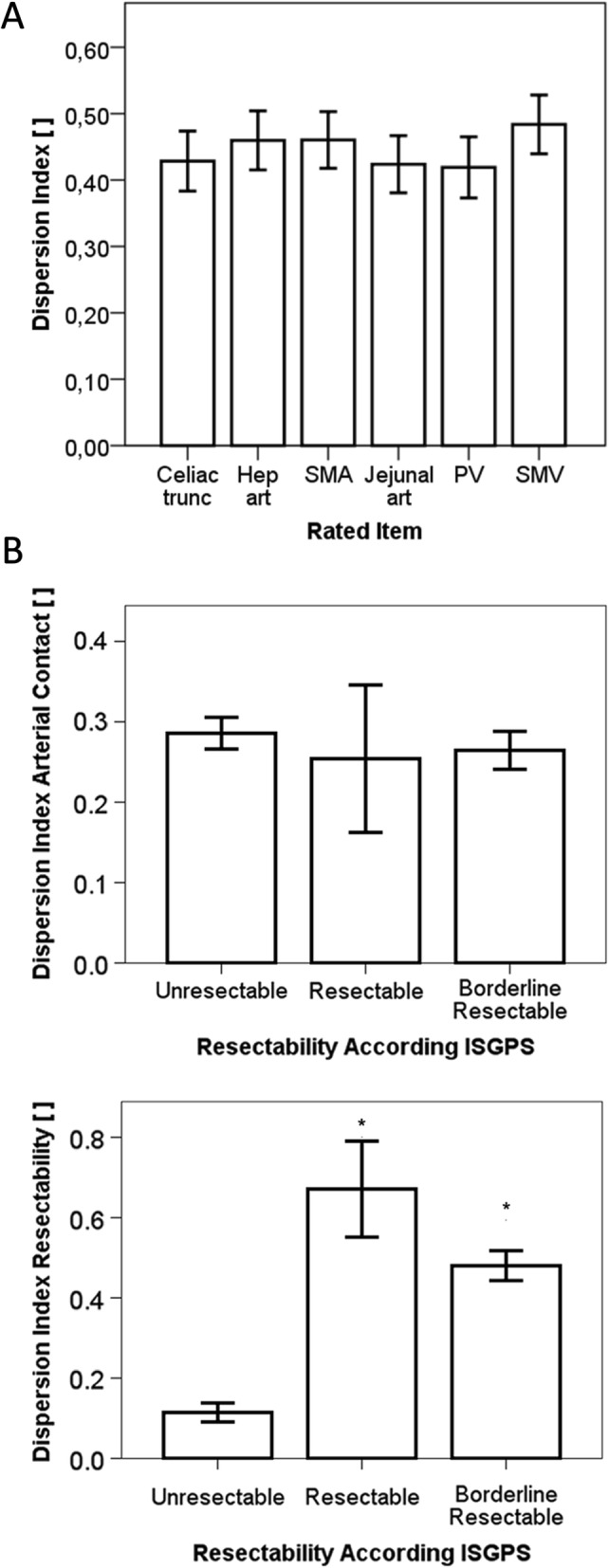
Dispersion indices of the parameters evaluated in the individual patients. This dispersion index is a measure of homogeneity of judgement of one parameter in individual patients by several observers. Zero describes a perfect match of all observers. **a** When the index of dispersion was calculated for the individual vessels evaluated by the surgeons, the dispersion of evaluated tumor contact was not different between the vessels. **b** To evaluate the influence of the degree of tumor contact to the peripancreatic vascular structures, cases were classified for their anatomical resectability in resectable, borderline resectable and locally advanced. The average of the dispersion index of tumor contact to the vasculature was similar in patients with resectable, borderline resectable, and locally advanced tumors indicating the degree of tumor contact does not influence the observation of tumor abutment to vessels. **c** Using the same classification, analyses of the dispersion index of the judgement of resectability indicated, however, that the homogeneity of the conclusion drawn from the observation of tumor contact to the blood vessels depended significantly on the degree of tumor abutment. Especially in patients with resectable and borderline resectable tumors, the heterogeneity in the judgement of resectability was significantly decreased (*P* < 0.05) indicating a gap between anatomical resectability and subjective judgement by the individual surgeon

The index of dispersion was calculated in order to analyze the influence of the individual cases on the homogeneity of judgement. Differences in the assessment of tumor abutment to peripancreatic blood vessels were independent of the evaluated blood vessels, since the average index of dispersion was not different (Fig. 2a). Furthermore, cases were classified according to the anatomical resectability in resectable, borderline resectable and locally advanced and the dispersion of judgement of tumor abutment to peripancreatic vessels was evaluated. While anatomical resectability did not influence the judgement of arterial abutment (Fig. 2a), the conclusion drawn from these observations was significantly influenced by anatomical resectability (Fig. 2b). A significantly more homogeneous judgement was obtained for clearly locally advanced cases, while cases with signs of borderline resectability or even resectability were associated with a much greater degree of variation in the judgement by experienced pancreatic surgeons. This showed that not the observation of the anatomical tumor contact per se but the interpretation of resectability in borderline resectable cases was responsible for the difference in the judgement of resectability by pancreatic surgeons.

## Discussion

Determining the resectability of pancreatic cancer by contrast-enhanced CT scan has a reported positive predictive value of only 81% [21]. Responsible for the misjudgment of resectability were mostly undetected metastases but anatomical classification systems may also not correlate with resectability as a subset of patients present with removable but anatomically locally advanced pancreatic cancer. In our study, we analyzed the judgement by surgeons of identical CT scans and found a subjectivity of judgement between the individual surgeons in rating the tumor R0 removable, being uncertain in terms of resectability and classifying a tumor as locally advanced. Even the use of a simple scoring system based on the evaluation of tumor contact to vascular structures yielded differences in the resultant judgement.

The evaluation was undertaken prospectively in a cohort of patients with borderline resectable or locally advanced ductal adenocarcinoma of the pancreas prior to neoadjuvant therapy. The patient cohort did not include clearly resectable patients and the vast majority of patients displayed tumor contact to the peripancreatic vasculature. Single factors for determining resectability were assessed and graded in a system adapted from recent guidelines developed by a panel of experts [12]. These guidelines use the evaluation of tumor contact to the arterial structures surrounding the pancreas. The degree of arterial contact is subdivided in less or more than 180° tumor abutment to the affected artery. This is based on the experience that in these patients, dissection along the arterial adventitia makes resection of the tumor technically possible, while with more extensive tumor abutment, infiltration of the arterial wall becomes more and more likely. Despite the technical resectability in patients with tumor abutment of less than 180°, these patients frequently are left with positive or close resection margins after primary tumor resection, which correlated with reduced survival in previous studies [22]. Not only is the chance for complete tumor resection reduced, we also found that the judgement of borderline resectability is heterogeneous due to the necessity of evaluating the degree of tumor contact to the arteries. Surgeons were aware of the close proximity of the tumor to the neighboring artery but describing the degree of tumor abutment induced additional uncertainty. This questions the practicability of suggestions to introduce even more sophisticated parameters of tumor abutment judgement which also appear to lack clinical meaning [23, 24].

The biggest factor reducing homogeneity in the evaluation of tumor contact to the peripancreatic vasculature is the definition of the tumor outline on CT scans. In a substantial number of patients, the tumor presents isoattenuation to the surrounding pancreatic tissue [25] and the definition of the boundary of the tumor is frequently based on secondary signs, such as pancreatic duct dilation or bile duct dilation and stenosis. In these cases, normal parenchyma separating the tumor from adjacent vessels cannot be judged at all. While arteries present with perivascular hypodense tissue, this is not observed with the peripancreatic veins, suggesting this as the mechanism for the reduced homogeneity in judgement observed for the portal vein and superior mesenteric vein. Additionally, most tumors are surrounded by an inflammatory and desmoplastic reaction and it is a matter of debate whether the peritumorous desmoplastic reaction which is visible by reducing the hypodense space around the superior mesenteric artery is to be considered as tumor contact. Opposing this view, some surgeons consider the increase in density surrounding the superior mesenteric artery as tumor-free desmoplastic reaction and biologically stroma contact is already associated with a substantial decrease in survival after primary tumor resection [26].

In our analysis, even though strong agreement in arterial abutment was observed, the agreement in the overall judgement of resectability was lower. This indicated that surgeons drew different conclusions from identical observations. These differences in the judgement of resectability are not limited to pancreatic cancer. Strong differences in the judgement of resectability have also been reported for the resection of liver metastasis of colorectal cancer [27, 28], despite the use of well-defined classification systems. Our data indicate that in order to increase the interobserver agreement in complex scoring systems, these systems should only include a limited number of clinically-relevant parameters and these parameters should not be subdivided in multiple categories if possible.

## Conclusion

To develop reliable and reproducible detection systems of resectability, our data indicate that the assessment of the degree of tumor contact is of critical importance, especially in patients with tumor contact to arteries. Future studies will have to determine if the differentiation between tumor contact and tumor encasement has the necessary clinical impact. Despite these differences, surgeons showed strong agreement in detecting tumor contact to arterial structures, which is the most important factor determining resectability. The conclusion drawn from these observations require further clarification of the oncological meaning of the degree of tumor contact to the peripancreatic vasculature.

## Data Availability

Additional data are available from the corresponding author on request.

## Abbreviations

CT: Computer tomography
DICOM: Digital Imaging and Communications in Medicine
ICH-GCP: International Conference on Harmonisation-Good Clinical Practice
ISGP: International study group for pancreatic cancer
MRI: Magnetic resonance imaging

## References

[1] Vincent A, Herman J, Schulick R, Hruban RH, Goggins M (2011). Pancreatic cancer. Lancet Lond Engl.

[2] Li D, Xie K, Wolff R, Abbruzzese JL (2004). Pancreatic cancer. Lancet Lond Engl.

[3] Neoptolemos JP, Stocken DD, Dunn JA (2001). Influence of resection margins on survival for patients with pancreatic cancer treated by adjuvant chemoradiation and/or chemotherapy in the ESPAC-1 randomized controlled trial. Ann Surg.

[4] McGuigan A, Kelly P, Turkington RC, Jones C, Coleman HG, McCain RS (2018). Pancreatic cancer: a review of clinical diagnosis, epidemiology, treatment and outcomes. World J Gastroenterol.

[5] Gilbert JW, Wolpin B, Clancy T (2017). Borderline resectable pancreatic cancer: conceptual evolution and current approach to image-based classification. Ann Oncol Off J Eur Soc Med Oncol.

[6] Soweid AM (2017). The borderline resectable and locally advanced pancreatic ductal adenocarcinoma: definition. Endosc Ultrasound.

[7] Lopez NE (2014). Borderline resectable pancreatic cancer: definitions and management. World J Gastroenterol.

[8] Katz MHG, Pisters PWT, Evans DB (2008). Borderline resectable pancreatic cancer: the importance of this emerging stage of disease. J Am Coll Surg.

[9] Varadhachary GR, Tamm EP, Abbruzzese JL (2006). Borderline resectable pancreatic cancer: definitions, management, and role of preoperative therapy. Ann Surg Oncol.

[10] Callery MP, Chang KJ, Fishman EK, Talamonti MS, William Traverso L, Linehan DC (2009). Pretreatment assessment of resectable and borderline resectable pancreatic cancer: expert consensus statement. Ann Surg Oncol.

[11] Tempero MA, Arnoletti JP, Behrman SW (2012). Pancreatic adenocarcinoma, version 2.2012: featured updates to the NCCN guidelines. J Natl Compr Cancer Netw JNCCN.

[12] Bockhorn M, Uzunoglu FG, Adham M (2014). Borderline resectable pancreatic cancer: a consensus statement by the international study group of pancreatic surgery (ISGPS). Surgery.

[13] Mollberg N, Rahbari NN, Koch M, Hartwig W, Hoeger Y, Büchler MW, Weitz J (2011). Arterial resection during pancreatectomy for pancreatic cancer: a systematic review and meta-analysis. Ann Surg.

[14] Loizou L, Albiin N, Ansorge C (2013). Computed tomography staging of pancreatic cancer: a validation study addressing interobserver agreement. Pancreatology.

[15] Mazzola R, Fersino S, Aiello D (2018). Linac-based stereotactic body radiation therapy for unresectable locally advanced pancreatic cancer: risk-adapted dose prescription and image-guided delivery. Strahlenther Onkol.

[16] Dobiasch S, Goerig NL, Fietkau R, Combs SE (2018). Essential role of radiation therapy for the treatment of pancreatic cancer: novel study concepts and established treatment recommendations. Strahlenther Onkol.

[17] Bachmayer S, Fastner G, Vaszi A (2018). Nonmetastatic pancreatic cancer: improved survival with chemoradiotherapy > 40 Gy after systemic treatment. Strahlenther Onkol.

[18] Prokosch H-U, Ries M, Beyer A (2011). IT infrastructure components to support clinical care and translational research projects in a comprehensive cancer center. Stud Health Technol Inform.

[19] García-pérez MA, Núñez-antón V (2003). Cellwise residual analysis in two-way contingency tables. Educ Psychol Meas.

[20] Loether HJ, MacTavish DG (1976). Descriptive statistics for sociologists: an introduction. 3. print.

[21] Somers I, Bipat S (2017). Contrast-enhanced CT in determining resectability in patients with pancreatic carcinoma: a meta-analysis of the positive predictive values of CT. Eur Radiol.

[22] Hartwig W, Gluth A, Hinz U (2016). Outcomes after extended pancreatectomy in patients with borderline resectable and locally advanced pancreatic cancer. Br J Surg.

[23] Loyer EM, David CL, Dubrow RA, Evans DB, Charnsangavej C (1996). Vascular involvement in pancreatic adenocarcinoma: reassessment by thin-section CT. Abdom Imaging.

[24] Li H, Zeng MS, Zhou KR, Jin DY, Lou WH (2005). Pancreatic adenocarcinoma: the different CT criteria for peripancreatic major arterial and venous invasion. J Comput Assist Tomogr.

[25] Prokesch RW, Chow LC, Beaulieu CF, Bammer R, Jeffrey RB (2002). Isoattenuating pancreatic adenocarcinoma at multi-detector row CT: secondary signs. Radiology.

[26] Wellner UF, Krauss T, Csanadi A (2016). Mesopancreatic stromal clearance defines curative resection of pancreatic head cancer and can be predicted preoperatively by radiologic parameters: a retrospective study. Medicine (Baltimore).

[27] Mohammad WM, Martel G, Mimeault R, Fairfull-Smith RJ, Auer RC, Balaa FK (2012). Evaluating agreement regarding the resectability of colorectal liver metastases: a national case-based survey of hepatic surgeons. HPB.

[28] Folprecht G, Gruenberger T, Bechstein WO (2010). Tumour response and secondary resectability of colorectal liver metastases following neoadjuvant chemotherapy with cetuximab: the CELIM randomised phase 2 trial. Lancet Oncol.

